# Editorial: Molecular Genetics of Cutaneous Melanoma: Current Status and Future Direction

**DOI:** 10.3389/fmolb.2021.811341

**Published:** 2021-12-22

**Authors:** Cristina Pellegrini, Zaida Garcia-Casado, William C. Cho

**Affiliations:** ^1^ Dermatology, Department of Biotechnological and Applied Clinical Sciences, University of L’Aquila, L’Aquila, Italy; ^2^ Labortory of Molecular Biology, Instituto Valenciano de Oncologia, Valencia, Spain; ^3^Department of Clinical Oncology, Queen Elizabeth Hospital, Hong Kong SAR, China

**Keywords:** biomarker, BRAF-mutated melanoma, cutaneous melanoma, somatic, mutation

Cutaneous melanoma is considered to be one of the most aggressive and treatment-resistant cancers. Although new therapies are currently available for metastatic disease, survival for patients with advanced disease is still poor.

Melanoma pathogenesis is complex and heterogeneous in genetics, with the involvement of genetic, environmental, and host factors. It arises from the transformation of melanocytes through the accumulation of genetic abnormalities at the somatic level involving critical signaling pathways (such as MAPK, PI3K/AKT, pRB/p16INK4, and p53) and it shows the highest mutation rate among all cancers (Cancer Genome Atlas Network, 2015). Recently, a molecular classification (including the BRAF-, NRAS-, NF1-mutated, and triple wild-type melanoma subgroups) has been proposed according to the genetic candidate drivers (Shain and Bastian, 2016). However, this still fails to fully address the molecular complexity of melanoma, which is also driven by trascriptomic and epigenetic changes alterations and/or by genomic rearrangements. The identification of tumor-specific genetic alterations in melanoma is currently one of the most active areas of melanoma research. Several clinical trials using novel therapies targeting known dysregulated signaling pathways are ongoing.

Thanks to new next-generation sequencing (NGS) strategies that allow the identification of novel emerging molecular pathways by high-throughput sequencing analysis of the large regions of the human genome, significant advances in melanoma genetics have been achieved. We envision this research topic will draw attention to the current knowledge and potential future prospective of the molecular genetics of cutaneous melanoma.

As reviewed by Vanni et al., BRAF mutation testing has become a priority to determine the choice and course of therapy. The authors discussed the recent molecular biology-based strategies for BRAF mutation detection, highlighting the advantages and disadvantages of the most commonly used diagnostic strategies and the timing of such molecular assessment. They suggested a diagnostic algorithm that included a sequential analysis of two methods, i.e. initial detection of V600E positive cases by immunohistochemistry together with molecular diagnostics (such as Sanger sequencing, pyrosequencing, real-time PCR-based, or NGS). Finally, an overview of the future strategies for molecular diagnosis was discussed, mainly focusing on the liquid biopsy. For BRAF-mutated melanoma, the advent of highly specific target drugs radically changes the therapeutic approach to melanoma and improves outcomes. Tanda et al. traced back the development of molecular target molecules until the current therapeutic scenario, starting from the introduction of BRAF inhibitors as a single agent to modern clinical practice that included the combination of BRAF plus MEK inhibitors as well as the use of targeted therapies in the adjuvant setting.

There is increasing interest in the molecular characterization of melanoma, aiming to identify additional molecular markers either targetable by new drugs or useful for prognosis. Vanni et al. reviewed the published studies involving more than 30 genes other than BRAF, which might become targets for new molecular targeted therapies. They reported the mutation data of 992 melanoma samples analyzed by whole-exome sequencing or whole-genome sequencing to demonstrate the genetic heterogeneity of melanoma beyond BRAF mutation. They also provided an overview of related clinical trials (completed or ongoing) to better outline the state of molecular-derived clinical research. Besides, Ying et al. collected the clinical and whole-exome sequencing data from two clinical cohorts of melanoma patients treated with immunotherapy. They demonstrated that the presence of somatic FSIP2 gene mutations might influence the response to treatment. Furthermore, their results showed that FSIP2 mutated melanomas had higher immunogenicity and fewer immunosuppressive Tregs cells in the microenvironment, therefore were more sensitive to immune checkpoint inhibitors. Rapanotti et al. identified a specific gene signature (including MCAM/MUC18/CD146/ABCB5, MMP, and VE-Cadh) that could define more aggressive circulating melanoma subpopulation cells. Additional studies would be useful to validate the prognostic role of these genes. Recently, Feng et al. examined the expression levels of the 17 most important RNA methylation regulators in the TCGA-SKCM dataset. According to the multivariate Cox regression analysis, three of them (ELF3, ZC3H13, and WTAP) were demonstrated to be potential prognostic factors in cutaneous melanoma.

Although the majority of melanomas occur sporadically, around 5–10% have a hereditary component (Rossi et al.). Germline susceptibility is associated with the mutations in high-penetrance melanoma predisposition gene CDKN2A and less frequently in CDK4, BAP1, TERT, and POT1 genes or with variants in intermediate-risk genes MC1R and MITF. New genome-wide association studies identified several low-risk loci with a role in melanoma development. In this context, De Summa et al. investigated the germline mutational profile of 32 genes that might contribute to the development of multiple primary melanoma. They found that the mutations in PIK3CA and CYP1B1 might contribute to the differential development of sporadic when compared to multiple melanoma.

In summary, the collection in this research topic embrace the current knowledge of the molecular aspects of melanoma pathogenesis, which are useful for the diagnostics and may suggest new tumor-specific alterations as prognostic biomarkers.
